# Endometrial compaction in patients with chronic endometritis does not predict pregnancy outcomes: a retrospective study

**DOI:** 10.3389/fendo.2025.1554201

**Published:** 2025-05-19

**Authors:** Qingmei Jin, Zhengao Sun, Zizhen Guo

**Affiliations:** ^1^ The First Clinical College, Shandong University of Traditional Chinese Medicine, Jinan, Shandong, China; ^2^ Department of Reproduction and Genetics, The Affiliated Hospital of Shandong University of Traditional Chinese Medicine, Jinan, Shandong, China

**Keywords:** endometrial compaction, chronic endometritis, endometrial receptivity, pregnancy outcomes, frozen-thawed embryo transfer

## Abstract

**Background:**

Endometrial compaction is known to positively influence pregnancy outcomes in patients with infertility undergoing assisted reproductive technology. However, the impact of endometrial compaction on pregnancy outcomes in patients with chronic endometritis following antibiotic treatment is not well understood.Considering the unique characteristics of patients with chronic endometritis, we aimed to investigate whether endometrial compaction predicts pregnancy outcomes during frozen-thawed embryo transfer cycles in this population.

**Methods:**

We enrolled 769 patients who underwent hysteroscopy before frozen-thawed embryo transfer over the past 3 years and subsequently categorized them into the populations with and without chronic endometritis to analyze the impact of endometrial compaction on pregnancy outcomes. To adjust for potential confounding factors, a multifactor binary logistic regression analysis was conducted using clinical co-variables that may have impacted the cycle results.

**Results:**

In patients with chronic endometritis following antibiotic treatment, endometrial compaction was not significantly associated with biochemical pregnancy (adjusted odds ratio [AOR] = 1.020, 95% confidence interval [CI] = 0.639–1.627), clinical pregnancy (AOR = 1.028, 95% CI = 0.643–1.643), ectopic pregnancy (AOR = 2.003, 95% CI = 0.145–27.635), early pregnancy loss (AOR = 1.356, 95% CI = 0.502–3.663), ongoing pregnancy (AOR = 0.970, 95% CI = 0.600–1.569), and live birth (AOR = 0.881, 95% CI 0.545–1.423). However, endometrial compaction was related to higher rates of clinical pregnancy (AOR = 1.623, 95% CI = 1.052–2.504), ongoing pregnancy (AOR = 2.193, 95% CI = 1.403–3.429), and live birth (AOR = 2.244, 95% CI = 1.431–3.520) in patients without chronic endometritis.

**Conclusion:**

Endometrial compaction was associated with improved pregnancy outcomes in patients without chronic endometritis but did not predict pregnancy outcomes in those with chronic endometritis, even after antibiotic treatment, suggesting that other factors influence pregnancy success in this population.

## 1 Introduction

Endometrial compaction is associated with favorable pregnancy outcomes in frozen-thawed embryo transfers (FETs) and is acknowledged as an indicator of endometrial receptivity. Researchers are increasingly investigating its relationship with the outcomes of *in vitro* fertilization (IVF); however, the results have been controversial. Haas et al. (1) and Zilberberg et al. (2) first proposed the concept of endometrial compaction and reported that patients with endometrial compaction demonstrated significantly higher rates of ongoing pregnancy than those with no change or an increase in endometrial thickness (EMT). Multiple studies have reported similar conclusions (3–5). However, some studies have demonstrated no correlation between endometrial compaction and pregnancy outcomes (6–9) or even opposing results (10). A recent systematic review and meta-analysis indicated no significant association between endometrial compaction under progesterone and live birth rates (LBRs) following embryo transfer, regardless of the type of embryo transfer or endometrial preparation protocol (11).

Changes in EMT following progesterone exposure are an indication of the degree of endometrial response to progesterone. During the follicular phase, estrogen secreted by the follicle stimulates continuous proliferation of the endometrial glands and blood vessels, thereby increasing EMT. Subsequently, further growth and curving of the endometrial glands and blood vessels are induced under the action of estrogen and progesterone during the luteal phase. Density, rather than thickness or volume, increases with the further development of glands and blood vessels. The continued growth of EMT during the luteal phase may be attributed to progesterone receptor deficiency or progesterone resistance, indicating an unfavorable environment for embryo transfer (1).

Chronic endometritis (CE) is an inflammatory disease of the endometrium that is typically caused by microbial disturbances in the uterine cavity and alterations in the immune environment (12, 13). With the popularization of hysteroscopy and pathological examinations, an increasing number of infertile women have been diagnosed with CE. CE is closely associated with repeated implantation failures (RIFs) (14) and recurrent spontaneous abortions (RSAs) (15). However, the effect of CE on embryo implantation and the related mechanisms remain inconclusive. Inflammatory environments alter the expression of progesterone targets in endometrial stromal cells (ESCs), which may lead to the progesterone resistance and a suboptimal local response to progestin therapy (16). A previous study found that CE could modify decidualization via the aberrant tuning of sex steroids and their receptors, and weaken the action of progesterone on ESCs resulting in a diminished potential for differentiation and an enhanced potential for proliferation of ESCs, similar to exposed to estradiol alone (17). Thus, inadequate endometrial compaction may be associated with CE. Currently, CE remains a challenge in clinical treatment, and empirical antibiotic treatments are commonly used. Studies have reported increased pregnancy rates among patients with CE following antibiotic treatment (18, 19).

Based on the aforementioned series of studies, we hypothesized that the unique characteristics of the population with CE account for the currently reported controversial conclusions regarding the relationship between endometrial compaction and pregnancy outcomes. Therefore, we aimed to investigate the predictive value of endometrial compaction on pregnancy outcomes in patients with or without CE, thereby supplementing and extending the current knowledge on this topic.

## 2 Methods

### 2.1 Study design and population

**Figure 1:**
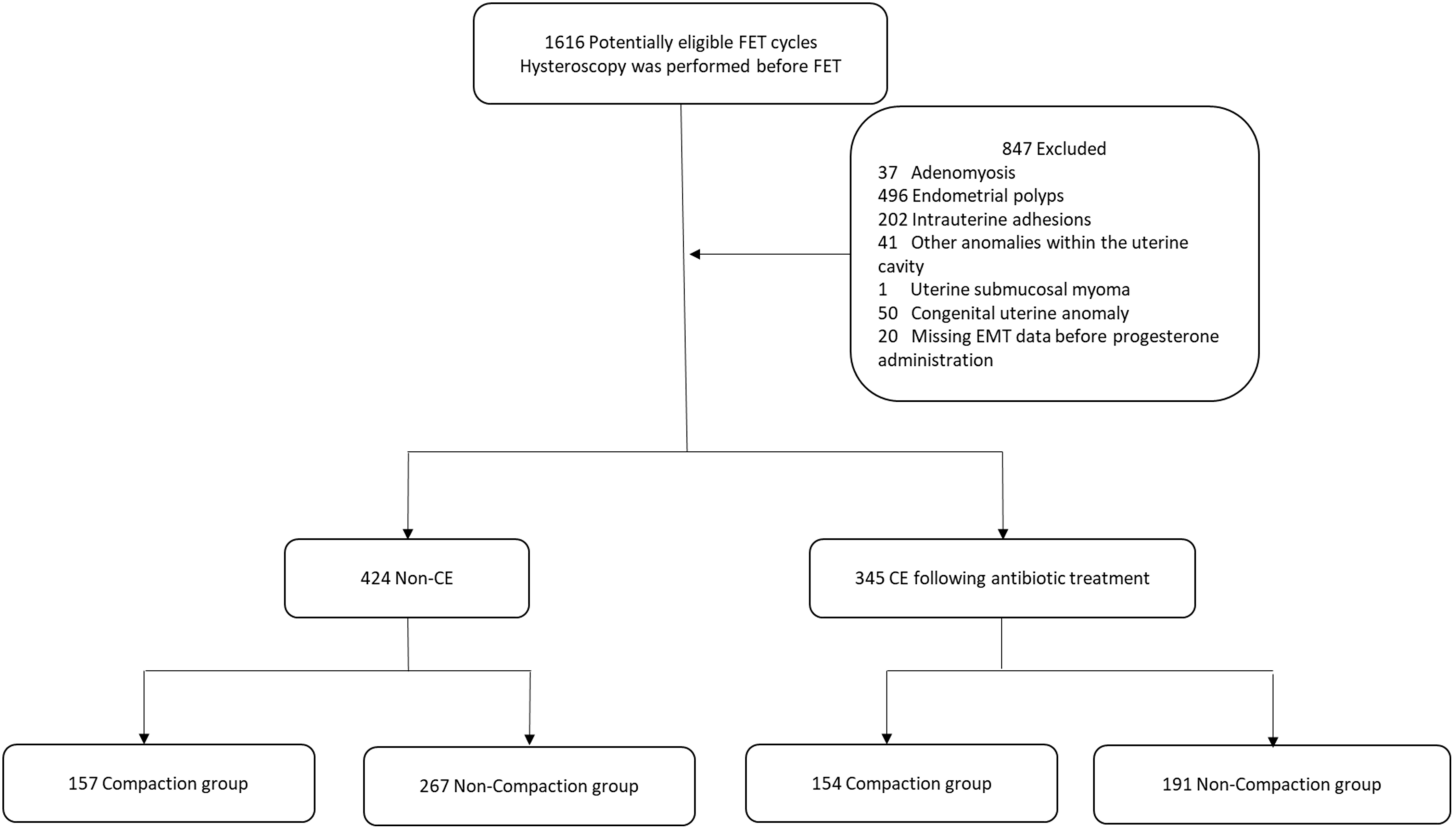
Flowchart of patients. FET, frozen-thawed embryo transfer; EMT, endometrial thickness; CE, chronic endometritis.

In this single-center retrospective cohort study, we collected clinical data from patients undergoing FET who had hysteroscopy at the Department of Reproduction and Genetics in the Affiliated Hospital of Shandong University of Traditional Chinese Medicine between January 1, 2020, and December 31, 2023. The eligibility criteria were patients with or without CE and undergoing FET cycles. In contrast, the exclusion criteria were hysteroscopy showing abnormalities in the uterine cavity, including endometrial polyps, intrauterine adhesions, and other anomalies within the uterine cavity; adenomyosis; uterine submucosal myoma; congenital uterine anomaly; and missing EMT data before progesterone administration. In total, 769 FET cycles were performed. We categorized the patients into those with CE (n = 345) and those without CE (n = 424). CE was diagnosed based on hysteroscopy (20) and immunohistochemistry (IHC) staining results for CD38 and CD138, with a criterion of ≥5 plasma cells/10 high-power fields in endometrial specimens (21). All patients with CE were orally administered doxycycline hydrochloride tablets (100 mg two times/day) (22). Endometrial compaction includes a reduction in the EMT from the end of the estrogen phase to the day of embryo transfer, whereas an uncompacted state is characterized by EMT remaining unchanged or thickening (9). Based on EMT changes, we further categorized patients of the non-CE and post-antibiotic CE groups into the following four subgroups: a compaction (n = 157) and non-compaction (n = 267) group of patients without CE, and a compaction (n = 154) and a non-compaction (n = 191) group of patients with CE following antibiotic treatment. **Figure 1** shows the flowchart of these categories. The study protocol was performed in accordance with the Code of Ethics of the World Medical Association (Declaration of Helsinki) and was approved by the Ethics Committee of the Affiliated Hospital of Shandong University of Traditional Chinese Medicine (approval number is (2024) Ethics Review No (087). -KY).

### 2.2 Endometrial preparation protocol

Based on the individual circumstances of the patients, endometrial preparation protocols included natural, ovulation induction, and hormone replacement treatment (HRT) cycles with or without gonadotropin-releasing hormone analog pretreatment. During the ovulation cycles, follicular development, EMT, and endometrial patterns were detected using transvaginal ultrasonography (TVUS) on days 10–12 of menstruation. Serum luteinizing hormone, estradiol, and progesterone were measured to determine the day of ovulation when the follicle diameter was ≥16 mm. Intramuscular progesterone (40 mg/day; Zhejiang Xianju Pharmaceutical Co., Ltd., Zhejiang, China) and oral dydrogesterone tablets (20 mg two times/day, Abbott Laboratories Trading [Shanghai] Co., Ltd., Shanghai, China) were added to the protocol following ovulation. Patients were administered 2.5–5 mg/day of letrozole tablets (LE, Zhejiang Haizheng Pharmaceutical Co., Ltd., Zhejiang, China), or not, for 5 days on days 3–5 of menstruation, as well as human menopausal gonadotropins (Shanghai Lizhu Pharmaceutical Co., Ltd., Shanghai, China) based on their actual condition.

During the HRT cycles, the patients were orally administered 4–8 mg/day estradiol valerate tablets (Delpharm Lille S.A.S.) for 12 days or more on days 2–5 of menstruation or progesterone withdrawal bleeding. When the EMT was ≥8 mm, 40 mg/day of intramuscular progesterone and 20 mg two times/day of oral dydrogesterone tablets were added for endometrial transformation. Participants undergoing HRT–FET with pituitary downregulation were subcutaneously injected with 3.75 mg of leuprolide acetate microspheres (Shanghai Lizhu Pharmaceutical Co., Ltd., Shanghai, China) on days 2–3 of menstruation. Estradiol valerate tablets were orally administered on days 28–30 following injection, and subsequent medication usage was similar to that of the HRT cycles. At the end of the estrogen phase, EMT was measured using TVUS, and on the day of embryo transfer, EMT was measured using abdominal ultrasonography (2, 5).

Based on the Istanbul Consensus and Gardner’s evaluation methods, we classified the quality of cleavage-stage embryos and blastocysts. High-quality embryos were defined as those at cleavage stage I and blastocysts graded 3BB or higher.

### 2.3 CE diagnosis

Each woman underwent a diagnostic office hysteroscopy during the follicular phase. The presence of endometrial hyperemia, micropolyps, or endometrial interstitial edema during hysteroscopy can serve as the basis for CE diagnosis (20). The criterion for CE diagnosis was hysteroscopy combined with the histological findings of multiple plasmacyte infiltration into the endometrial stroma, corresponding to IHC staining for CD38 and CD138. We preferred the diagnostic criterion of ≥5 plasma cells/10 high-power fields in endometrial specimens (21). Patients diagnosed with CE were administered 100 mg of doxycycline hydrochloride tablets orally, twice daily for a duration of 14 days (22). Repeat hysteroscopy and endometrial biopsy were not performed following antibiotic treatment (21). Previous studies have demonstrated that the presence or absence of endometrial re-evaluation has no impact on pregnancy outcomes in patients with CE after doxycycline treatment (23).

### 2.4 Definition of pregnancy outcomes

Live birth was the primary indicator of pregnancy outcomes in our study, whereas the secondary indicators were biochemical, clinical, ectopic, and ongoing pregnancies and early pregnancy loss. Biochemical pregnancy was defined as ≥10 U/L of serum human chorionic gonadotrophin detected 14 days following embryo transfer. Clinical pregnancy was defined as the presence of at least one gestational sac, as detected in ultrasonography images of the uterine cavity. Ectopic pregnancy was defined as at least one gestational sac, as detected in ultrasonography images outside the uterine cavity. Early pregnancy loss was defined as a spontaneous abortion before 12 weeks of intrauterine gestation. Ongoing birth was defined as an intrauterine gestation of up to 12 weeks. Live birth was defined as the delivery of at least one live baby after 28 weeks of gestation.

### 2.5 Statistical analysis

Data analysis was performed using IBM SPSS Statistics for Windows, version 26.0 (IBM Corp., Armonk, N.Y., USA). We analyzed the baseline characteristics of patients with or without CE using the Student’s t-test, Mann–Whitney U-test, and chi-squared test. Normality tests were performed on all continuous variables. Normally distributed continuous variables were expressed as mean ± standard deviation and were analyzed using the Student’s t-test. The Mann–Whitney U-test was used for non-normally distributed continuous variables, which were expressed as median and interquartile range. For qualitative (categorical) data, the chi-squared test was used to analyze the differences between the compaction and non-compaction groups. Statistical significance was set at P < 0.05.

Univariate and multivariate binary logistic regression analyses were used to compare pregnancy outcomes between the compaction and non-compaction groups, and between the CE and non-CE groups. The odds ratio and 95% confidence intervals (CIs) were calculated. To adjust for potential confounding factors, a multifactor binary logistic regression analysis was conducted using clinical co-variables that may have affected pregnancy outcomes, including maternal age at oocyte retrieval (24), endometrial preparation protocol (25), menstrual cycle (26), infertility type (26), infertility factor (27), body mass index (25), the time interval between the last hysteroscopy and FET (28), number of transferred embryos (25), number of high-quality transferred embryos (25), embryo type (29), and embryo source fertilization method (30). When comparing pregnancy outcomes between patients with and those without CE, an additional confounding factor of groups of EMT change ratio was added.

## 3 Results

### 3.1 Baseline characteristics of the patients

Among the patients who achieved clinical pregnancy, 14 patients experienced mid-term and late miscarriages.

We analyzed the basic characteristics of infertility in women with or without CE (in which patients with CE were treated with antibiotics) (**Table 1**). We observed significant differences regarding infertility duration among patients without CE and body mass index among patients with CE between endometrial compaction and non-compaction groups (all P < 0.05). Among patients diagnosed with CE who received antibiotic treatment, those with endometrial compaction transferred a greater number of high-quality embryos (P < 0.05). Additionally, EMT was thicker on the day of embryo transfer in the population with an uncompacted endometrium, regardless of the presence or absence of CE (P < 0.05).

**Table 1:** Baseline characteristics of the study population.

Characteristics	Non-CE (n=424)			CE (n=345)		
	Compaction(n=157)	Non-Compaction(n=267)	P value	Compaction (n=154)	Non-Compaction(n=191)	P value
Maternal age at oocyte retrieval, median (IQR), y	33 (29.0-37.0)	33 (29.0-36.0)	0.987	32 (29.0-35.2)	32 (29.0-36.0)	0.646
Maternal age at embryo transfer, median (IQR), y	33 (30.0-37.0)	33 (30.0-37.0)	0.672	32 (30.0-36.0)	33 (30.0-37.0)	0.550
Endometrial preparation protocol, No. (%)			0.350			0.591
Ovulation cycle	66 (42.0)	100 (37.5)		73 (47.4)	85 (44.5)	
HRT	91 (58.0)	167 (62.5)		81 (52.6)	106 (55.5)	
Menstrual cycle, No. (%)			0.494			0.105
Regularity	112 (71.3)	182 (68.2)		91 (59.1)	129 (67.5)	
Irregularity	45 (28.7)	85 (31.8)		63 (40.9)	62 (32.5)	
Infertility duration, median (IQR), y	3 (2–6)	3 (1–4)	0.001	3 (1–5)	3 (2– 6)	0.697
Infertility type, No. (%)			0.533			0.550
Primary	54 (34.4)	84 (31.5)		62 (40.3)	83 (43.5)	
Secondary	103 (65.6)	183 (68.5)		92 (59.7)	108(56.5)	
Infertility factor, No. (%)			0.402			0.374
Polycystic ovary syndrome	28 (17.8)	36 (13.5)		44 (28.6)	40 (20.9)	
Endometriosis	8 (5.1)	18 (6.7)		6 (3.9)	6 (3.1)	
Ovarian hypoplasia	2 (1.3)	8 (3.0)		6 (3.9)	7 (3.7)	
Tubal or male factor	119 (75.8)	205 (76.8)		98 (63.6)	138 (72.3)	
BMI, median (IQR), kg/m^2^	23.80 (21.35-25.60)	22.60 (20.90-25.60)	0.152	24.45 (21.30-27.20)	22.90 (21.00-25.60)	0.012
Time interval between the last hysteroscopy and FET, No. (%)			0.734			0.988
<3 months	34 (21.7)	50 (18.7)		53 (34.4)	65 (34.0)	
3–6 months	19 (12.1)	31 (11.6)		45 (29.2)	55 (28.8)	
>6 months	104 (66.2)	186 (69.7)		56 (36.4)	71 (37.2)	
Number of transferred embryos, No. (%)			0.716			0.145
1	61 (38.9)	99 (37.1)		65 (42.2)	66 (34.6)	
2	96 (61.1)	168 (62.9)		89 (57.8)	125 (65.4)	
Number of high-quality transferred embryos, median (IQR)	1 (0-1)	1 (0-1)	0.404	1 (0-1)	0 (0-1)	<0.001
Embryo type, No. (%)			0.679			0.283
Cleavage- stage embryo	98 (62.4)	172 (64.4)		88 (57.1)	120 (62.8)	
Blastocyst	59 (37.6)	95 (35.6)		66 (42.9)	71 (37.2)	
Embryo source fertilisation method, No. (%)			0.058			0.236
IVF	120 (76.4)	224 (83.9)		128 (83.1)	149 (78.0)	
ICSI	37 (23.6)	43 (16.1)		26 (16.9)	42 (22.0)	
EMT at embryo transfer day, median (IQR), mm	9.0 (8.0-10.0)	10.1 (9.0-12.0)	<0.001	9.0 (8.0-10.0)	11.0 (10.0-12.0)	<0.001
Baseline FSH, median (IQR), IU/l	6.87 (5.84-8.09)	7.01 (5.69-8.32)	0.881	6.46 (5.15-7.80)	6.46 (5.39-8.04)	0.607
Baseline LH, median (IQR), mIU/ml	4.52 (3.52-6.00)	4.70 (3.26-6.84)	0.593	4.98 (3.79-8.14)	4.95 (3.75-6.89)	0.509
Baseline E2, median (IQR), pg/ml	36.00 (26.01-48.22)	36.30 (27.00-49.68)	0.676	35.00 (26.07-44.39)	35.42 (26.00-53.33)	0.366
Baseline progesterone, median (IQR), ng/ml	0.46 (0.29-0.76)	0.46 (0.28-0.77)	0.695	0.39 (0.24-0.62)	0.46 (0.30-0.65)	0.146

CE, chronic endometritis; HRT, hormone replacement treatment; BMI, body mass index; IVF, *in vitro* fertilization; ICSI, intracytoplasmic sperm injection; EMT, endometrial thickness; FSH, follicle-stimulating hormone; LH, luteinizing hormone; E2, estradiol; IQR, interquartile range.

### 3.2 Pregnancy outcomes of patients without CE

We assessed the effect of endometrial compaction on pregnancy outcomes in patients without CE (**Table 2**). The clinical pregnancy rate (CPR), ongoing pregnancy rate (OPR), and LBR were higher in patients with endometrial compaction based on the univariate binary logistic regression analysis (all P < 0.05). However, no statistically significant differences were observed in the biochemical pregnancy rate (BPR), ectopic pregnancy rate (EPR), and early pregnancy loss rate (EPLR) between the two groups (all P > 0.05). The multivariate binary logistics regression analysis revealed that the CPR (adjusted odds ratio [AOR] = 1.623, 95% CI = 1.052–2.504), OPR (AOR = 2.193, 95% CI = 1.403–3.429), and LBR (AOR = 2.244, 95% CI = 1.431–3.520) in the compaction group were still higher than those in the non-compaction group.

**Table 2:** Comparison of pregnancy outcomes between endometrial compaction and non-compaction groups in patients without CE.

Outcome	Compaction	Non-compaction	Univariate analysis ^a^		Multivariate analysis ^b^	
	n=157	n=267	OR (95% CI)	P value	OR (95% CI)	P value
Biochemical pregnancy, No. (%)	95 (60.5)	144 (53.9)	1.309 (0.877-1.953)	0.188	1.343 (0.872-2.071)	0.181
Clinical pregnancy, No. (%)	79 (50.3)	103 (38.6)	1.613 (1.083-2.402)	0.019	1.623 (1.052-2.504)	0.029
Ectopic pregnancy, No. (%)	2 (1.3)	3 (1.1)	1.135 (0.188-6.870)	0.890	1.671 (0.225-12.397)	0.615
Early pregnancy loss, No. (%)	11 (7.0)	31 (11.6)	0.574 (0.280-1.176)	0.129	0.483 (0.227-1.030)	0.060
Ongoing pregnancy, No. (%)	68 (43.3)	72 (27.0)	2.069 (1.366-3.135)	0.001	2.193 (1.403-3.429)	0.001
Live birth, No. (%)	66 (42.0)	68 (25.5)	2.122 (1.395-3.230)	<0.001	2.244 (1.431-3.520)	<0.001

CI, confidence interval; CE, chronic endometritis; OR, odds ratio.

a No adjustments for other covariates.

b Adjustment for maternal age at oocyte retrieval, endometrial preparation protocol, menstrual cycle, infertility type, infertility factor, BMI, time interval between the last hysteroscopy and FET, number of transferred embryos, number of high-quality transferred embryos, embryo type, embryo source fertilisation method.

### 3.3 Pregnancy outcomes of patients with CE following antibiotic treatment

**Table 3** presents the effect of endometrial compaction on pregnancy outcomes in patients with CE following antibiotic treatment, revealing no statistically significant differences between endometria with and without compaction in terms of BPR, CPR, EPR, EPLR, OPR, and LBR based on the univariate binary logistic regression analysis (all P > 0.05). The multivariate binary logistic regression analysis showed similar results, where no statistically significant differences were observed in BPR (AOR = 1.020, 95% CI = 0.639–1.627), CPR (AOR = 1.028, 95% CI = 0.643–1.643), EPR (AOR = 2.003, 95% CI = 0.145–27.635), EPLR (AOR = 1.356, 95% CI = 0.502–3.663), OPR (AOR = 0.970, 95% CI = 0.600–1.569), and LBR (AOR = 0.881, 95% CI = 0.545–1.423) between the compaction and non-compaction groups. Notably, all CIs encompassed the null value (1.0), indicating insufficient evidence to reject the null hypothesis of no association.

**Table 3:** Comparison of pregnancy outcomes between endometrial compaction and non-compaction groups in patients with CE following antibiotic treatment.

Outcome	Compaction	Non-compaction	Univariate analysis ^a^		Multivariate analysis ^b^	
	n=154	n=191	OR (95% CI)	P value	OR (95% CI)	P value
Biochemical pregnancy, No. (%)	86 (55.8)	98 (51.3)	1.200 (0.784-1.838)	0.401	1.020 (0.639-1.627)	0.935
Clinical pregnancy, No. (%)	72 (46.8)	82 (42.9)	1.167 (0.762-1.789)	0.478	1.028 (0.643-1.643)	0.910
Ectopic pregnancy, No. (%)	3 (1.9)	1 (0.5)	3.775 (0.389-36.657)	0.252	2.003 (0.145-27.635)	0.604
Early pregnancy loss, No. (%)	9 (5.8)	10 (5.2)	1.123 (0.445-2.838)	0.806	1.356 (0.502-3.663)	0.548
Ongoing pregnancy, No. (%)	63 (40.9)	72 (37.7)	1.144 (0.741-1.767)	0.543	0.970 (0.600-1.569)	0.902
Live birth, No. (%)	57 (37.0)	70 (36.6)	1.016 (0.654-1.577)	0.944	0.881 (0.545-1.423)	0.604

CE, chronic endometritis; CI: confidence interval; OR: odds ratio

a No adjustments for other covariates

b Adjustment for maternal age at oocyte retrieval, endometrial preparation protocol, menstrual cycle, infertility type, infertility factor, BMI, time interval between the last hysteroscopy and FET, number of transferred embryos, number of high-quality transferred embryos, embryo type, embryo source fertilisation method.

### 3.4 Pregnancy outcomes between patients with and those without CE (in which patients with CE were treated with antibiotics)

The univariate and multivariate binary logistic regression analyses revealed a higher rate of early pregnancy loss (9.9% vs 5.5% and P < 0.05) in patients without CE than those with CE (**Table 4**). However, no statistically significant associations were observed between the CE and non-CE patients regarding the BPR (AOR = 0.738, 95% CI = 0.534–1.019), CPR (AOR = 0.823, 95% CI = 0.593–1.142), EPR (AOR = 1.182, 95% CI = 0.278–5.029), OPR (AOR = 1.006, 95% CI = 0.718–1.409), or LBR (AOR = 0.970, 95% CI = 0.691–1.360) after multivariate binary logistic regression analyse. Notably, all non-significant CIs encompassed the null value (1.0), indicating insufficient evidence to reject the null hypothesis of no association.

**Table 4:** Comparison of pregnancy outcomes between patients with or without CE (in which CE patients were treated with antibiotics).

Outcome	Non-CE	CE	Univariate analysis ^a^		Multivariate analysis ^b^	
	n=424	n=345	OR (95% CI)	P value	OR (95% CI)	P value
Biochemical pregnancy, No. (%)	239 (56.4)	184 (53.3)	0.885 (0.665-1.177)	0.400	0.738 (0.534-1.019)	0.065
Clinical pregnancy, No. (%)	182 (42.9)	154 (44.6)	1.072 (0.805-1.428)	0.634	0.823 (0.593-1.142)	0.245
Ectopic pregnancy, No. (%)	5 (1.2)	4 (1.2)	0.983 (0.262-3.689)	0.980	1.182 (0.278-5.029)	0.821
Early pregnancy loss, No. (%)	42 (9.9)	19 (5.5)	0.530 (0.302-0.930)	0.027	0.495 (0.269-0.910)	0.024
Ongoing pregnancy, No. (%)	140 (33.0)	135 (39.1)	1.304 (0.970-1.754)	0.079	1.006 (0.718-1.409)	0.973
Live birth, No. (%)	134 (31.6)	127 (36.8)	1.261 (0.934-1.701)	0.130	0.970 (0.691-1.360)	0.859

CE, chronic endometritis; CI, confidence interval; OR, odds ratio.

a No adjustments for other covariates.

b Adjusted for maternal age at oocyte retrieval, endometrial preparation protocol, menstrual cycle, infertility type, infertility factor, BMI, time interval between the last hysteroscopy and FET, number of transferred embryos, number of high-quality transferred embryos, embryo type, embryo source fertilisation method, groups of EMT change ratio.

### 3.5 Pregnancy outcomes between the CE and non-CE groups in patients with endometrial compaction

We compared the pregnancy outcomes between CE and non-CE groups in patients with endometrial compaction (**Table 5**). No statistically significant differences regarding the BPR, CPR, EPR, EPLR, OPR, or LBR were found between the two groups based on the univariate binary logistic regression analysis (All P > 0.05). Notably, in patients with endometrial compaction, the BPR (AOR = 0.560, 95% CI = 0.328–0.956), CPR (AOR = 0.574, 95% CI = 0.339–0.974), OPR (AOR = 0.585, 95% CI = 0.344–0.996), and LBR (AOR = 0.534, 95% CI = 0.312–0.912) in the non-CE group were higher than those in the CE group after correcting for confounding factors in the multivariate binary logistics regression analysis.

**Table 5:** Comparison of pregnancy outcomes between non-CE and CE following antibiotic treatment groups in patients with endometrial compaction.

Outcome	Non-CE	CE following antibiotic treatment	Univariate analysis ^a^		Multivariate analysis ^b^	
	n=157	n=154	OR (95% CI)	P value	OR (95% CI)	P value
Biochemical pregnancy, No. (%)	95 (60.5)	86 (55.8)	0.825 (0.526-1.296)	0.404	0.560 (0.328-0.956)	0.033
Clinical pregnancy, No. (%)	79 (50.3)	72 (46.8)	0.867 (0.556-1.353)	0.529	0.574 (0.339-0.974)	0.040
Ectopic pregnancy, No. (%)	2 (1.3)	3 (1.9)	1.540 (0.254-9.344)	0.639	1.054 (0.121-9.209)	0.962
Early pregnancy loss, NO. (%)	11 (7.0)	9 (5.8)	0.824 (0.331-2.047)	0.677	0.950 (0.341-2.647)	0.922
Ongoing pregnancy, No. (%)	68 (43.3)	63 (40.9)	0.906 (0.578-1.422)	0.668	0.585 (0.344-0.996)	0.048
Live birth, No. (%)	66 (42.0)	57 (37.0)	0.810 (0.514-1.278)	0.365	0.534 (0.312-0.912)	0.022

CI, confidence interval; CE, chronic endometritis; OR, odds ratio.

a No adjustments for other covariates.

b Adjusted for maternal age at oocyte retrieval, endometrial preparation protocol, menstrual cycle, infertility type, infertility factor, BMI, time interval between the last hysteroscopy and FET, number of transferred embryos, number of high-quality transferred embryos, embryo type, embryo source fertilization method.

## 4 Discussion

For the first time, we categorized patients based on the presence or absence of CE and arrived at the following conclusions (1): Endometrial compaction was associated with better pregnancy outcomes in patients without CE (2). However, in those with CE, endometrial compaction did not serve as a reliable predictor of pregnancy outcomes, even after standard antibiotic treatment.

The relationship between CE and female infertility has attracted widespread attention from clinicians, particularly in the field of assisted reproduction. CE affects endometrial decidualization and the expression of immune cells and cytokines, which are detrimental to embryo implantation and affect pregnancy outcomes. Moreover, CE is closely related to RIFs and RSAs. Among infertile women with RIFs, one-third were diagnosed with CE and experienced a significant improvement in their LBR after antibiotic treatment (22). A retrospective study revealed that CE increases the miscarriage rate and decreases the LBR and term birth rate in patients undergoing IVF (31). McQueen et al. (15) found that CE prevalence was high in women with RSAs, with over 50% positive for CD138 plasma cells detected using IHC. Furthermore, this study indicated that women with untreated CE exhibited a tendency toward a higher miscarriage rate and lower LBR than women without CE, but these differences were not significant between women with and those without CE.

Fertility in women with RSAs could be restored using the appropriate antibiotic treatment, suggesting that CE may be a cause of RSAs (32). Studies have indicated that antibiotics are an effective way to treat CE and improve pregnancy outcomes in patients with CE. however, the potential advantages of CE treatment for embryo implantation and the progression of pregnancy remain controversial. One study reported that the number of early pregnancy losses in patients with CE was higher than that in those without CE, even after being cured with antibiotics, suggesting that antibiotic-cured CE was still a risk factor for early pregnancy loss (33). Recently, a large prospective cohort study confirmed that patients with antibiotic-cured CE had a higher risk of spontaneous abortion and that the LBR was significantly lower than that in patients without CE following subsequent IVF/intracytoplasmic sperm injection (34).

We found that patients with CE experienced pregnancy outcomes comparable to those without CE subsequent to antibiotic treatment, but the EPLR was significantly higher than that in patients without CE. The patients did not undergo a biopsy for embryonic aneuploidy before embryo transfer, and we did not exclude patients with embryonic chromosomal abnormalities when calculating the EPLR, which may explain why our outcomes are inconsistent with those of earlier research. However, due to the absence of embryonic aneuploidy data, we are unable to comprehensively assess the effects of relevant variables. Tus, this hypothesis requires validation through future studies.

Among the 769 FET cycles we investigated, none of the embryos underwent preimplantation genetic testing for aneuploidies, and embryo quality might have had an effect on pregnancy outcomes. A previous study demonstrated that implantation of high-quality euploid blastocysts assessed using pre-implantation genetic testing for aneuploidies and morphology did not improve live birth outcomes in IVF (35). Thus, endometrial receptivity remains a critical condition for embryo implantation. However, a definitive indicator of endometrial receptivity is yet to be identified. Our results indicated that endometrial compaction could predict pregnancy outcomes in patients with a healthy and non-inflammatory endometrium. Endometrial compaction is associated with a higher rate of clinical pregnancy, ongoing pregnancy, and live birth. Haas et al. (1) found that endometrial compaction following progesterone administration could improve the OPR in women via single-blastocyst transplantation in HRT–FETs, where certain blastocysts were subjected to aneuploidy biopsies. Subsequently, researchers from the same center analyzed the data of patients undergoing euploid blastocyst transplantation (2). Their results were similar to ours. Youngster et al. (4) also demonstrated that the CPR and OPR were higher in the endometrial compaction group in unstimulated natural cycle FETs, and 46% of patients presented >5% endometrial compaction without progesterone administration. In contrast, Jin et al. (10) found that an increase in EMT following progesterone administration was associated with a higher CPR in the natural cycles of euploid blastocyst transfer. However, only 25.1% of the endometrial compaction rates were reported by Jin et al. (10). Serum progesterone levels do not affect endometrial development during the secretory period (36). Therefore, endometrial compaction is a critical factor in pregnancy outcomes in addition to embryonic factors.

To our knowledge, this is the first study to discuss the relationship between endometrial compaction and pregnancy outcomes in patients with CE following antibiotic treatment during FET cycles. We standardized the antibiotic treatment for all patients when they were diagnosed with CE using hysteroscopy and IHC. Notably, patients with CE following antibiotic treatment had significantly improved pregnancy outcomes, with an LBR similar to that in those without CE. However, among patients with endometrial compaction, pregnancy outcomes were superior in patients without CE than in those with CE, even when those with CE received antibiotic treatment. The lack of predictive power of endometrial compaction may reflect irreversible endometrial damage or incomplete resolution of CE, rather than being attributed to the endometrial compaction itself. Long-term inflammation may lead to endometrial fibrosis (37, 38) or vascular lesions (39) in patients with CE, which cannot be directly and completely reversed by antibiotic treatment. These structural alterations of endometrium may impair its function and hinder embryo implantation and placental development. In addition, CE is frequently accompanied with alterations of immune microenvironment, including changes of types and numbers of immune cells within endometrium, as well as abnormalities of cytokine and chemokine levels (40). And excessive release of pro-inflammatory cytokines may damage embryo through pro-apoptotic and adverse programming effects, as well as indirectly suppressing uterine receptivity through the maternal immune response (41). The imbalance of immune microenvironment induced by CE may not be rapidly restored following antibiotic treatment. Recently, studies have demonstrated the existence of diverse microbial communities within endometrial environment. Alterations of microbiome in endometrium may impact female fertility. Compared to healthy women, the endometrial microbiome in patients with CE was significantly altered, exhibiting a marked reduction of the abundance of Lactobacillus (42). Moreno et al. (43) observed that women with an endometrial microbiome dominated by Lactobacillus tend to have better pregnancy outcomes. Although antibiotics can effectively inhibit pathogenic bacteria, they are unable to restore the abundance of beneficial microorganisms, such as Lactobacillus, and may disrupt the normal composition of the reproductive tract microbiota.

The strengths of this study include real-world data presentation, the relatively large number of cycles investigated, all patients being from the same center, and virtually all endometrial preparation protocols being covered. Therefore, the results of this study are broadly applicable to women undergoing FETs. However, this study had certain limitations. Owing to the retrospective nature of this investigation, not all confounding factors could be properly controlled. We attempted to use a multivariate logistic regression analysis in an effort to adjust for as many potential confounding factors as possible. The study design and data collection were based on existing clinical records and treatment protocols, which may not have all ideal follow-up examinations, including repeat hysteroscopy and endometrial biopsy. Some unmeasured confounders, such as antibiotic treatment adherence and microbial resistance, may limit the ability to draw causal inferences. To address this limitation, future studies should consider a prospective design with large sample size and include regular hysteroscopy after treatment to better evaluate the effects of antibiotic therapy on patients with CE, thereby further strengthening the scientific validity of the research outcomes. Additionally, EMT was measured using TVUS at the end of the estrogen phase and abdominal ultrasonography on the day of embryo transfer. Generally, EMT is more accurately measured using TVUS. Although, all ultrasound examinations were performed by experienced ultrasound technicians from the same center, and any potential measurement bias was evenly distributed among all patients, the variation of measurements from estrogen phase to transfer day may impact the results of study. Future studies should mitigate through consistent TVUS utilization at all assessment timepoints.

## 5 Conclusion

We discussed how progesterone-induced EMT alterations affect pregnancy outcomes in patients with CE following antibiotic treatment. Endometrial compaction in patients with CE following antibiotic treatment could not predict pregnancy outcomes. In the future, larger samples and prospective cohort studies are required to validate our findings and further explore whether other potential factors affect pregnancy outcomes in patients with CE following antibiotic treatment.

## Data Availability

The raw data supporting the conclusions of this article will be made available by the authors, without undue reservation.

## References

[1] Haas J Smith R Zilberberg E Nayot D Meriano J Barzilay E. Endometrial compaction (decreased thickness) in response to progesterone results in optimal pregnancy outcome in frozen-thawed embryo transfers. Fertility Sterility. (2019) 112:503–509.e1. doi: 10.1016/j.fertnstert.2019.05.001 31248618

[2] Zilberberg E Smith R Nayot D Haas J Meriano J Barzilay E. Endometrial compaction before frozen euploid embryo transfer improves ongoing pregnancy rates. Fertility Sterility. (2020) 113:990–5. doi: 10.1016/j.fertnstert.2019.12.030 32386621

[3] Bu Z Yang X Song L Kang B Sun Y. The impact of endometrial thickness change after progesterone administration on pregnancy outcome in patients transferred with single frozen-thawed blastocyst. Reprod Biol Endocrinol. (2019) 17:99. doi: 10.1186/s12958-019-0545-0 31767010 PMC6876076

[4] Youngster M Mor M Kedem A Gat I Yerushalmi G Gidoni Y. Endometrial compaction is associated with increased clinical and ongoing pregnancy rates in unstimulated natural cycle frozen embryo transfers: a prospective cohort study. J Assist Reprod Genet. (2022) 39:1909–16. doi: 10.1007/s10815-022-02544-7 PMC9428085 35727423

[5] Ju W Wei C Lu X Zhao S Song J Wang H. Endometrial compaction is associated with the outcome of artificial frozen-thawed embryo transfer cycles: a retrospective cohort study. J Assist Reprod Genet. (2023) 40:1649–60. doi: 10.1007/s10815-023-02809-9 PMC10352224 37140828

[6] Jin Z Shi H Lu M Bu Z Huo M Zhang Y. Endometrial thickness changes after progesterone administration do not affect the pregnancy outcomes of frozen-thawed euploid blastocyst transfer: a retrospective cohort study. Fertility Sterility. (2021) 116:1502–12. doi: 10.1016/j.fertnstert.2021.08.008 34538461

[7] Riestenberg C Quinn M Akopians A Danzer H Surrey M Ghadir S. Endometrial compaction does not predict live birth rate in single euploid frozen embryo transfer cycles. J Assist Reprod Genet. (2021) 38:407–12. doi: 10.1007/s10815-020-02043-7 PMC7884553 33389380

[8] Olgan S Dirican EK Sakinci M Caglar M Ozsipahi AC Gul SM. Endometrial compaction does not predict the reproductive outcome after vitrified–warmed embryo transfer: a prospective cohort study. Reprod BioMedicine Online. (2022) 45:81–7. doi: 10.1016/j.rbmo.2022.02.025 35501270

[9] Shah JS Vaughan DA Dodge LE Leung A Korkidakis A Sakkas D. Endometrial compaction does not predict live birth in single euploid frozen embryo transfers: a prospective study. Hum Reprod. (2022) 37:980–7. doi: 10.1093/humrep/deac060 35357436

[10] Jin Z Li J Yang E Shi H Bu Z Niu W. Effect of endometrial thickness changes on clinical pregnancy rates after progesterone administration in a single frozen-thawed euploid blastocyst transfer cycle using natural cycles with luteal support for PGT-SR- and PGT-M-assisted reproduction: a retrospective cohort study. Reprod Biol Endocrinol. (2021) 19:154. doi: 10.1186/s12958-021-00841-x 34627292 PMC8501735

[11] Feng S Wang B Chen S Xie Q Yu L Xiong C. Association between proliferative-to-secretory endometrial compaction and pregnancy outcomes after embryo transfer: a systematic review and meta-analysis. Hum Reprod. (2024) 39:749–59. doi: 10.1093/humrep/deae012 38323525

[12] Chen P Chen P Guo Y Fang C Li T. Interaction between chronic endometritis caused endometrial microbiota disorder and endometrial immune environment change in recurrent implantation failure. Front Immunol. (2021) 12:748447. doi: 10.3389/fimmu.2021.748447 34671363 PMC8521098

[13] Chu M He S Zhao H Yin S Liu Z Zhang W. Increasing expression of STING by ERα antagonizes LCN2 downregulation during chronic endometritis. J Reprod Immunol. (2023) 160:104167. doi: 10.1016/j.jri.2023.104167 37952294

[14] Li Y Xu Y Yu S Lin S Chen W Lian R. Chronic endometritis impairs embryo implantation in patients with repeated implantation failure: A retrospective study. Taiwanese J Obstetrics Gynecology. (2022) 61:984–8. doi: 10.1016/j.tjog.2021.01.034 36428002

[15] McQueen DB Perfetto CO Hazard FK Lathi RB. Pregnancy outcomes in women with chronic endometritis and recurrent pregnancy loss. Fertility Sterility. (2015) 104:927–31. doi: 10.1016/j.fertnstert.2015.06.044 26207958

[16] Grandi G Mueller MD Papadia A Kocbek V Bersinger NA Petraglia F. Inflammation influences steroid hormone receptors targeted by progestins in endometrial stromal cells from women with endometriosis. J Reprod Immunol. (2016) 117:30–8. doi: 10.1016/j.jri.2016.06.004 27371899

[17] Wu D Kimura F Zheng L Ishida M Niwa Y Hirata K. Chronic endometritis modifies decidualization in human endometrial stromal cells. Reprod Biol Endocrinol. (2017) 15:16. doi: 10.1186/s12958-017-0233-x 28259137 PMC5336610

[18] Xiong Y Chen Q Chen C Tan J Wang Z Gu F. Impact of oral antibiotic treatment for chronic endometritis on pregnancy outcomes in the following frozen-thawed embryo transfer cycles of infertile women: a cohort study of 640 embryo transfer cycles. Fertility Sterility. (2021) 116:413–21. doi: 10.1016/j.fertnstert.2021.03.036 33926717

[19] Li J Li X Ding J Zhao J Chen J Guan F. Analysis of pregnancy outcomes in patients with recurrent implantation failure complicated with chronic endometritis. Front Cell Dev Biol. (2023) 11:1088586. doi: 10.3389/fcell.2023.1088586 36861040 PMC9969095

[20] Song D Li T-C Zhang Y Feng X Xia E Huang X. Correlation between hysteroscopy findings and chronic endometritis. Fertility Sterility. (2019) 111:772–9. doi: 10.1016/j.fertnstert.2018.12.007 30683588

[21] Bouet P-E El Hachem H Monceau E Gariépy G Kadoch I-J Sylvestre C. Chronic endometritis in women with recurrent pregnancy loss and recurrent implantation failure: prevalence and role of office hysteroscopy and immunohistochemistry in diagnosis. Fertility Sterility. (2016) 105:106–10. doi: 10.1016/j.fertnstert.2015.09.025 26456229

[22] Kitaya K Matsubayashi H Takaya Y Nishiyama R Yamaguchi K Takeuchi T. Live birth rate following oral antibiotic treatment for chronic endometritis in infertile women with repeated implantation failure. Am J Rep Immunol. (2017) 78:e12719. doi: 10.1111/aji.12719 28608596

[23] Liu W Huang J Sun L Huang L Zhang Q Nong Y. New biopsy after antibiotic treatment: effect on outcomes of assisted reproduction in patients with infertility and chronic endometritis. Reprod BioMedicine Online. (2022) 45:1167–75. doi: 10.1016/j.rbmo.2022.07.020 36462787

[24] Mattei G Reschini M Li Piani L Fornelli G Vigano P Muzii L. Unexplained infertility and age-related infertility: indistinguishable diagnostic entities but different IVF prognosis. Hum Reprod. (2024) 39:1996–2002. doi: 10.1093/humrep/deae140 38906837

[25] Veleva Z Orava M Nuojua-Huttunen S Tapanainen JS Martikainen H. Factors affecting the outcome of frozen-thawed embryo transfer. Hum Reprod. (2013) 28:2425–31. doi: 10.1093/humrep/det251 23756705

[26] Xu B Hou Z Liu N Zhao J Li Y. Pretreatment with a long-acting GnRH agonist for frozen-thawed embryo transfer cycles: how to improve live birth? J Ovarian Res. (2023) 16:197. doi: 10.1186/s13048-023-01277-0 37743479 PMC10518919

[27] Siristatidis C Pouliakis A Sergentanis TN. Special characteristics, reproductive, and clinical profile of women with unexplained infertility versus other causes of infertility: a comparative study. J Assist Reprod Genet. (2020) 37:1923–30. doi: 10.1007/s10815-020-01845-z PMC7467999 32504303

[28] Wang B Meng N Zhang W Kong P Liu Z Liu W. Optimal waiting period for frozen embryo transfer after hysteroscopic polypectomy: A propensity score matching analysis. Front Endocrinol. (2022) 13:986809. doi: 10.3389/fendo.2022.986809 PMC9561364 36246905

[29] Dirican EK Olgan S Sakinci M Caglar M. Blastocyst versus cleavage transfers: who benefits? Arch Gynecol Obstet. (2022) 305:749–56. doi: 10.1007/s00404-021-06224-2 34487220

[30] Abbas AM Hussein RS Elsenity MA Samaha II El Etriby KA Abd El-Ghany MF. Higher clinical pregnancy rate with *in-vitro* fertilization versus intracytoplasmic sperm injection in treatment of non-male factor infertility: Systematic review and meta-analysis. J Gynecology Obstetrics Hum Reprod. (2020) 49:101706. doi: 10.1016/j.jogoh.2020.101706 32018045

[31] Morimune A Kimura F Nakamura A Kitazawa J Takashima A Amano T. The effects of chronic endometritis on the pregnancy outcomes. Am J Rep Immunol. (2021) 85:e13357. doi: 10.1111/aji.13357 33020952

[32] Cicinelli E Matteo M Tinelli R Pinto V Marinaccio M Indraccolo U. Chronic endometritis due to common bacteria is prevalent in women with recurrent miscarriage as confirmed by improved pregnancy outcome after antibiotic treatment. Reprod Sci. (2014) 21:640–7. doi: 10.1177/1933719113508817 PMC3984485 24177713

[33] Zhang Q Yang G Tan J Xiong Y Xu Y Xu Y. Antibiotic cured chronic endometritis remains a risk factor for early pregnancy loss in the subsequent frozen euploid embryo transfer. Reprod BioMedicine Online. (2024) 48:103611. doi: 10.1016/j.rbmo.2023.103611 38118232

[34] Duan H Li X Hao Y Shi J Cai H. Risk of spontaneous abortion after antibiotic therapy for chronic endometritis before *in vitro* fertilization and intracytoplasmic sperm injection stimulation. Fertility Sterility. (2022) 118:337–46. doi: 10.1016/j.fertnstert.2022.04.026 35691723

[35] Ozgur K Berkkanoglu M Bulut H Yoruk GDA Candurmaz NN Coetzee K. Single best euploid versus single best unknown-ploidy blastocyst frozen embryo transfers: a randomized controlled trial. J Assist Reprod Genet. (2019) 36:629–36. doi: 10.1007/s10815-018-01399-1 PMC6505013 30617927

[36] Usadi RS Groll JM Lessey BA Lininger RA Zaino RJ Fritz MA. Endometrial development and function in experimentally induced luteal phase deficiency. J Clin Endocrinol Metab. (2008) 93:4058–64. doi: 10.1210/jc.2008-0460 PMC2729203 18647810

[37] Liu L Yang H Guo Y Yang G Chen Y. The impact of chronic endometritis on endometrial fibrosis and reproductive prognosis in patients with moderate and severe intrauterine adhesions: a prospective cohort study. Fertil Steril. (2019) 111:1002–1010.e2. doi: 10.1016/j.fertnstert.2019.01.006 30922643

[38] Radzinsky VE Kostin IN Petrov YA Polina ML Gasanova BM. Diagnostic significance of chronic endometritis macrotypes differentiation among women with reproductive losses. Gynecol Endocrinol. (2017) 33:36–40. doi: 10.1080/09513590.2017.1399697 29264986

[39] Carvalho FM Aguiar FN Tomioka R de Oliveira RM Frantz N Ueno J. Functional endometrial polyps in infertile asymptomatic patients: a possible evolution of vascular changes secondary to endometritis. Eur J Obstet Gynecol Reprod Biol. (2013) 170:152–6. doi: 10.1016/j.ejogrb.2013.05.012 23773528

[40] Zeng S Liu X Liu D Song W. Research update for the immune microenvironment of chronic endometritis. J Reprod Immunol. (2022) 152:103637. doi: 10.1016/j.jri.2022.103637 35576684

[41] Robertson SA Chin P-Y Femia JG Brown HM. Embryotoxic cytokines-Potential roles in embryo loss and fetal programming. J Reprod Immunol. (2018) 125:80–8. doi: 10.1016/j.jri.2017.12.003 29306096

[42] Liu Y Ko EY-L Wong KK-W Chen X Cheung W-C Law TS-M. Endometrial microbiota in infertile women with and without chronic endometritis as diagnosed using a quantitative and reference range-based method. Fertil Steril. (2019) 112:707–17.e1. doi: 10.1016/j.fertnstert.2019.05.015 31327470

[43] Moreno I Codoñer FM Vilella F Valbuena D Martinez-Blanch JF Jimenez-Almazán J. Evidence that the endometrial microbiota has an effect on implantation success or failure. Am J Obstet Gynecol. (2016) 215:684–703. doi: 10.1016/j.ajog.2016.09.075 27717732

